# Co-occurring congeners coordinate drought and shade tolerance traits along gradients of canopy openness

**DOI:** 10.1007/s00442-026-05950-1

**Published:** 2026-08-10

**Authors:** Sam W. Anderson, Jackson Cramer, Katherine A. McCulloh

**Affiliations:** University of Wisconsin – Madison Botany Department, 430 Lincoln Dr, Madison, WI 53706 USA

**Keywords:** Trait syndrome, Acclimation, Irradiance, Stress tolerance

## Abstract

Physiological traits related to water status and photosynthesis biophysically link plant performance to environmental conditions like light and water availability. By approaching the concept of species’ niches from a trait-based perspective, physiological traits can be used to characterize how trait syndromes diverge to limit direct resource competition and promote resource partitioning in sympatric species. Such traits could also inform broader studies of changing community composition in an era of drastic shifts in climate or disturbance regimes. However, the extent of inter/intraspecies trait variation and how such traits vary along local environmental gradients remains an open question in understanding physiological traits as species-level characteristics. To address this, we quantified hydraulic and gas exchange traits in four co-occurring *Viburnum* species while also quantifying local canopy coverage and soil conditions. All species displayed significant variation in gas-exchange traits, turgor loss point, and heavy carbon isotope discrimination along gradients of canopy openness. Specifically, plants in shadier environments exhibited higher photosynthetic rates at low light levels while individuals growing in gaps demonstrated higher drought tolerance and stricter stomatal regulation. Soil texture explained little variation in traits assessed. *V. acerifolium* displayed a more conservative, shade-tolerant trait syndrome, yet little significant difference was found in traits between the congeners, highlighting the potentially limited resolution of physiological traits to differentiate trait syndromes of closely related, co-occurring species.

**Supplementary Information:**

The online version contains supplementary material available at https://doi.org/10.1007/s00442-026-05950-1.

## Introduction

Identifying coordination patterns in the responses of plant traits to environmental gradients is a central line of inquiry in functional trait ecology (Kearney et al. 2010; Lavorel et al. 2011; Funk et al. 2017). In particular, physiological traits can provide insight into patterns of co-occurrence, resource partitioning, and adaptive cross-over in closely related species by quantifying divergence in trait syndromes (Cavender-Bares et al. 2004; Beck and Givnish 2021; Beck et al. 2022). As such, the increasing use of species-level resolution of physiological functional traits is central to studies of functional niche in community ecology, especially in sympatric systems (Nadal et al. 2023).

Traits quantifying fluxes associated with gas exchange during photosynthesis or water status and transport inherently tie plant metabolism to environmental conditions and are central to our understanding of plant fitness, survival, growth, and stress tolerance (Reich 2014; Funk et al. 2017; Chacón-Labella et al. 2023). Species-level means of physiological traits complement prevailing analyses more generally tied to ‘acquisitive’ and ‘conservative’ plant strategies by more directly quantifying carbon assimilation, transpiration, and acclimation to environmental conditions such as shade or water availability (Wright et al. 2004; Nadal et al. 2023; Streit and Bellwood 2023). As functional trait ecology continues to expand as a discipline, quantifying intraspecific trait variation is essential to improving our understanding of functional trait modelling. Particularly, intraspecific variation is key to addressing assumptions about how trait variance is partitioned within and between species or correlated with scale-dependent environmental parameters (Niinemets 2015; Anderegg et al. 2018; Westerband et al. 2021; Chacón-Labella et al. 2023).

Most terrestrial plants rely on water transported from the soil to maintain cellular function and replace water lost through transpiration. Terrestrial plants are well adapted to utilize available water by creating a continuous flow of water from soil to roots to leaves while balancing hydraulic vulnerability and conductivity driven by transpiration and declining water potential (Sperry 2003; Angeles et al. 2004). However, the availability of water, characterized by soil water field capacity, conductivity, and matric potential, is strongly influenced by soil texture (Campbell and Norman 1998). Provided that sampling occurs within geographically proximal sites with similar meteorological conditions, quantifying soil texture allows for direct comparison of species’ water use strategies in relation to water availability, fostering a more mechanistic understanding of the link between local conditions and plant water status regulation and acclimation.

Hydraulic traits have become increasingly useful in understanding the efficiency and limitations of water transport, especially in varying conditions of water availability (Sperry et al. 1988; Domec et al. 2009a, b; Mcculloh et al. 2014). Stem specific conductivity (K_S,_ g s^− 1^ MPa^− 1^ mm^− 1^) is a measure of water movement through stem tissue at a given pressure head. When adjusted using the total leaf area supported by that stem (A_L_A_S_), the leaf area adjusted stem conductivity (K_S, L_ g s^− 1^ m^− 1^ MPa^− 1^) provides insight into the hydraulic support for a given area of photosynthetic tissue. K_S_ and K_S, L_ have both been identified as key traits of variation between closely related species as they relate to water availability, and such traits can be readily integrated into an understanding of acquisitive vs. conservative trait syndromes (Reich 2014; Oliveira et al. 2021; Smith et al. 2023).

At the leaf level, turgor loss point (Ψ_TLP_, MPa) is the water potential at which leaf cells lose turgor, which has been linked to stomatal closure (Bartlett et al. 2014). Plants with high (less negative) Ψ_TLP_ typically close stomata and reduce photosynthesis during water-stressed conditions to limit transpiration, leading to their characterization as “drought-avoidant” (Brodribb et al. 2003; Salvi et al. 2022). Alternatively, species with lower (more negative) Ψ_TLP_ continue photosynthesis despite dry conditions, leading to their characterization as “drought-tolerant” (Bartlett et al. 2014; Salvi et al. 2021). With the adoption of more rapid protocols, larger analyses and datasets are being used to assess Ψ_TLP_ as a species-level trait (Bartlett et al. 2012; Maréchaux et al. 2015; Sjöman et al. 2018). However, many studies quantifying Ψ_TLP_ utilize only sun-grown leaves, limiting our understanding of species-level variation in Ψ_TLP_ (Eisley and Wolfe 2024). When combined with analyses of heavy carbon isotope discrimination (δ^13^C, VPDB), Ψ_TLP_ can inform how microsite variation indirectly impacts carbon fixation through regulation of stomatal conductance, assuming minimal differences in stomatal density and leaf N content. Because gradients in light and water availability are ubiquitous throughout nature, research dedicated to understanding how physiological traits are coordinated along local gradients in light and water availability would quantify key assumptions made about Ψ_TLP_, more realistically assess variation among sympatric species, and identify potential physiological divergence among congeners (McGregor et al. 2021).

The heterogenous light environment of forest understories begets variation in a key resource for photosynthetic organisms. Driving the carbon assimilation central to the growth and survival of plants, adaptations and acclimations to high-light and low-light environments have long been hypothesized and quantified (Givnish et al. 2004; Montgomery and Givnish 2008; Givnish and Montgomery 2014; Poorter et al. 2019). Adaptation to high irradiance involves increased allocation towards palisade parenchyma, higher leaf nitrogen concentration, higher leaf mass per area (LMA, g m^− 2^), and a higher saturated photosynthetic rate (A_max_, µmol m^− 2^ s^− 1^). This increased capacity for assimilation comes at the cost of higher dark respiration rates (R_d_, µmol m^− 2^ s^− 1^) and higher instantaneous leaf compensation points (LCP, µ mol m^− 2^ s^− 1^) (Givnish et al. 2004). Shade-grown plants are likely to have higher A_L_A_S_ and larger mean leaf area to maximize light interception, as well as lower LMA, A_max,_ LCP, and R_d_ to minimize costs associated with photosynthetic tissue. Assimilation compared on a unit leaf mass basis can also directly inform our understanding of photosynthetic ‘return on investment’ (Craine and Reich 2005; Valladares and Niinemets 2008; Valladares et al. 2016; Feng et al. 2018). Quantifying intraspecific variation in these traits across gradients in light and water availability would illuminate the scale-dependence of trait coordination in shade/drought tolerance, separating out patterns of species adaptation (niche partitioning) versus individual acclimation to light availability (niche overlap).

Temperate, deciduous forest systems exhibit environmental heterogeneity that provides a powerful framework with which to assess the impacts of varied light and soil substrate on plant physiology. Forests of southern Wisconsin, USA represent a well-studied cohort of communities united by similar climatic conditions while shaped broadly by heterogenous edaphic conditions shaped by glaciation, deposition, and erosion (Clayton and Moran 1982). Such variations in substrate can lead to dramatic variation in nutrient and water availability within short geographical distances (Curtis and McIntosh 1951; Leach and Givnish 1999; Rogers et al. 2008). Sites with abundant water and nutrient resources (i.e. silt/loam soils) also induce competition for light amongst existing vegetation, resulting in heterogenous light availability in forest understories (Curtis and McIntosh 1951). As a result, repeated regional ecological surveys have shown upland forest understory composition to be intimately connected to edaphic conditions related to soil texture and subsequent heterogeneity in understory light availability (Rogers et al. 2008; Amatangelo et al. 2011).

In deciduous temperate forests, the shrub layer is particularly well suited for studies of trait coordination and variation, especially since diverse genera like *Viburnum* allow us to identify divergent patterns in trait syndromes while limiting the effects of large phylogenetic distance on trait variation (Clement et al. 2014; Landis et al. 2021; Supplemental). In southern WI, four species of *Viburnum* (*V. acerifolium*,* V. lentago*,* V. trilobum*, and *V. rafinesquianum*) are all common in upland communities. These congeners often occupy similar habitats and grow throughout a range of forest types, though *V. acerifolium* is generally characterized as a more shade tolerant species (Barnes and Wagner 2004; Voss and Reznicek 2012). With qualitative support of varied ecology, a propensity towards sympatry, and documented ability to persist in varied light and soil conditions, these temperate *Viburnum* provide a model clade in which to assess in-situ physiological trait variation.

Considering the importance of identifying scale-dependent sources of variation of commonly measured physiological traits, our objective was to assess how traits related to water status and light response vary and co-vary within and between *Viburnum* species along local environmental gradients. Specifically, we set out to test whether gas exchange traits and traits related to hydraulic capacity (K_S_ and K_S, L_), drought tolerance (Ψ_TLP_) and stomatal regulation (δ^13^C, VPDB) would vary locally in accordance with the carbon-gain hypothesis identified in other studies of shade tolerance (Givnish and Montgomery 2014; Wright et al. 2004). Using a mixed-modelling approach and partial model R_lik_^2^ (Ives 2019) to quantify the variance explained by both gradients of interest and differences among congeners, while analysis of variance (ANOVA), principal components analysis (PCA), and trait-trait regressions were used to identify patterns of trait-coordination among and within *Viburnum* species.

Given descriptions of *V. acerifolium* occupying shadier habitats, we hypothesized that this species will have higher A_L_A_S_ and lower LMA, A_max_, LCP, and R_d_, than other congeners (Givnish 1988; Voss and Reznicek 2012). Across species, we hypothesized increasing LMA, LCP, A_max,_ R_d_, and hydraulic conductivity in more irradiated microsites in accordance with the carbon-gain hypothesis, while Ψ_TLP_ will decrease. Sandier soils will promote higher LMA and δ^13^C as well as lower hydraulic conductivity and Ψ_TLP_ to balance water loss and carbon gain in more water-limited growing conditions. The comprehensive result of these trait-environment relationships will be more shade-tolerant individuals in shaded, siltier microsites, while more drought-tolerant trait syndromes will be present in more open and/or sandier microsites, with *V. acerifolium* displaying a diverging trait syndrome from the other congeners.

## Materials and methods

### Study sites selection

Ten study sites were located within the Baraboo Hills and natural areas in Madison, WI, with a maximum distance between sites of 49 km (S2). The Baraboo Hills are an outcropping of quartzite that remained elevated despite repeated glacial advancement throughout the Holocene. Both the Baraboo Hills and Madison sites were significantly impacted by the erosive and depositional forces of glaciation, which caused high local heterogeneity in edaphic conditions, with varied soil textures, depths, and underlying bedrock (Dalziel et al. 1970). Specific sites of co-occurrence were identified from citizen science observations on iNaturalist and confirmed with preliminary site visits. Individuals, identified as distinct clusters of multiple ramets, were labelled for sampling based on the presence of healthy leaf tissue and sufficient aboveground biomass to allow for the sampling of multiple ramets. The twenty individuals sampled for each species were spread among sites based on co-occurrence and maximizing variation in local environmental conditions. During the summer of data collection (May – September 2023), field sites received a daily average of 2.5 mm rain fell over the and had an average daily temperature of 20.7 °C, relative humidity of 68%, and potential daily evapotranspiration of 4.83 mm (Bradford 2025).

### Soil and canopy analysis

At the base of each individual, a ~ 300 g soil sample was taken from the top 15 cm of mineral soil and analyzed following the soil texture protocols of the University of Wisconsin Soils and Forage Lab to provide a measure of percent clay, percent silt, and percent sand. Hemispheric photographs of the canopy were taken using a Google Pixel 4a spherical panorama located 1.25 m above the soil surface (Arietta 2022). To minimize direct solar interference, photos were taken in early morning and on overcast days. Spherical panoramas were converted to hemispheric photographs and binarized using ImageJ (Schneider et al. 2012). Binarized hemispheric photographs were verified to confirm that direct sunlight was not altering subsequent measurements. Canopy metrics were quantified using both the R package *HemispheR* (version 1.1.7) and ImageJ add-on Hemispheric2.0 to corroborate multiple metrics of gap structure (Chianucci and Cutini 2012; Beckschäfer 2015; Chianucci and Macek 2022).

### Trait data collection

Rapid light response curves were performed between 7:00am and 11:30am in July 2023 using a LiCOR-6800 Portable Photosynthesis Machine. Curves were fit using Q_in_ levels of 1800, 1500, 1000, 500, 250, 100, 50, 25, 15, and 0 µmol m^−2^s^− 1^_,_ with an ambient CO_2_ of 400 µmol m^−2^s^− 1^¸ flow rate of 500 µmol s^− 1^, leaf VPD of 1.5 kPa, and T_air_ of 25–27 °C. Curves were parameterized by non-rectangular hyperbola model using R package *photosynthesis* (version 2.1.5), allowing for the characterization of A_max_, LCP, and R_d_ on a leaf area basis (Marshall and Biscoe 1980; Stinziano et al. 2021, 2023). After curves had been completed, leaves were taken to calculate leaf mass per area (LMA) to allow for quantification of parameters on a leaf mass basis.

In July and August 2023, a healthy ramet ~1 cm in diameter was harvested pre-dawn from each individual, placed in an opaque bag, and transported to campus within 2 h. Five leaves were taken per individual for LMA analysis following standardized methods (Pérez-Harguindeguy et al. 2013). All leaves on a branch were placed in a 65° C oven to dry for 48 h to calculate bulk dry mass, which was then converted to bulk fresh area using LMA to calculate leaf area-to-sapwood area ratios (A_L_A_s_) (Pérez-Harguindeguy et al. 2013). Dry leaf tissue was processed via ball-mill and sent to Kansas State University’s Stable Isotope Mass Spectroscopy Lab, where percent Nitrogen (N), percent carbon (C), δ^13^C, and δ^15^N were analyzed.

To measure maximum hydraulic conductivity, stem segments were recut underwater with a razor blade, submerged in an aqueous solution of 20 m M KCl, and placed under a dynamic vacuum for 30 min to remove embolisms. Stem hydraulic conductivity was calculated as the flow rate the KCl solution through the stem onto an analytical balance divided by the gravitational pressure head inducing flow (Sperry et al. 1988). The open-source software *conductR* in R Studio (Smith 2018) was used to record flow rate to make calculations. Hydraulic conductivity was standardized by stem cross sectional area (stem-specific hydraulic conductivity; Ks, g s^− 1^ MPa^− 1^ mm^− 1^) and leaf area (K_S, L_. g s^− 1^ MPa^−1^m^− 1^). To calculate stem (D_stem_) and sapwood (D_sap_) density, approximately 1.5 cm segments of stem tissue were taken from stems used for conductivity and bark was removed. Sapwood density samples were split longitudinally, and pith tissue was removed. After vacuum infiltration, sample volumes were measured using volume displacement after which samples were dried for 48 h at a 65 C and dry mass measured.

### Statistical analyses

All statistical analyses were done in R (version 4.4.2), while all figures were created using R package *ggplot2* (version 2.3.0) (Wickham 2011; R Core Team 2025). Species-level differences in traits were analyzed using analysis of variance (ANOVA), with subsequent pairwise comparisons tested using Tukey’s Honest Significant Difference. Log transformed canopy parameters Leaf Area Index (LAI), gap-fraction, and gap area were highly correlated, as were the soil texture parameters percent silt, sand, and clay. As a result, models were constructed using gap-fraction to describe canopy structure and percent sand to characterize soil texture.

Linear mixed models were used to identify patterns in trait variation among species and in relation to environmental gradients using R package *lme4* (version 1.1–37). Leaf area and A_L_A_s_ were log-transformed to meet assumptions of normality. A mixed modelling approach was used with site as a random effect to account for an inherently hierarchical sampling structure. Nested model comparison was used to test a priori hypotheses of how traits vary between species, environmental factors and their interaction, using partial R_lik_^2^ to quantify model parsimony and variance explained by model parameters (Ives 2019). Model comparison using reduced models containing only species fixed effects and site random effects were used to calculate R_lik_^2^ using R package *rr2 *(Ives et al. 2019; Table 1). Species trait syndromes were descriptively characterized by principal components analysis (PCA), allowing for interpretation of trait syndromes between species in relation to light and soil texture. Trait coordination within species was also represented by trait correlation plots, displaying only significant trait-trait relationships between them as weighted links.

**Table 1:** The R_lik_^2^ of models explaining traits (columns) along gradients of gap fraction and sand. R_lik_^2^ are calculated using likelihood ratios while compared with the reduced model in row 1

Model	LMA	Ψ_TLP_	A_area_	A_mass_	A_L_A_S_	D_stem_	K_S, L_	LCP	δ^13^C	*N*
Trait~ Species+(1|Site)	0.00	0.00	0.00	0.00	0.00	0.00	0.00	0.00	0.00	0.00
Trait~ Species + Gap + (1|Site)	0.66	0.38	0.23	0.00	0.31	0.04	0.11	0.28	0.41	0.07
Trait~ Species + Sand + (1|Site)	0.01	0.00	0.01	0.01	0.00	0.02	0.00	0.02	0.01	0.00
Trait~ Species*Gap + (1|Site)	0.68	0.40	0.29	0.05	0.40	0.18	0.12	0.28	0.50	0.10
Trait~ Species*Sand + (1|Site)	0.04	0.01	0.03	0.05	0.02	0.10	0.03	0.07	0.01	0.08
Trait~ Species + Gap + Sand + (1|Site)	0.66	0.39	0.23	0.01	0.32	0.05	0.12	0.28	0.41	0.08

## Results

**Fig. 1:**
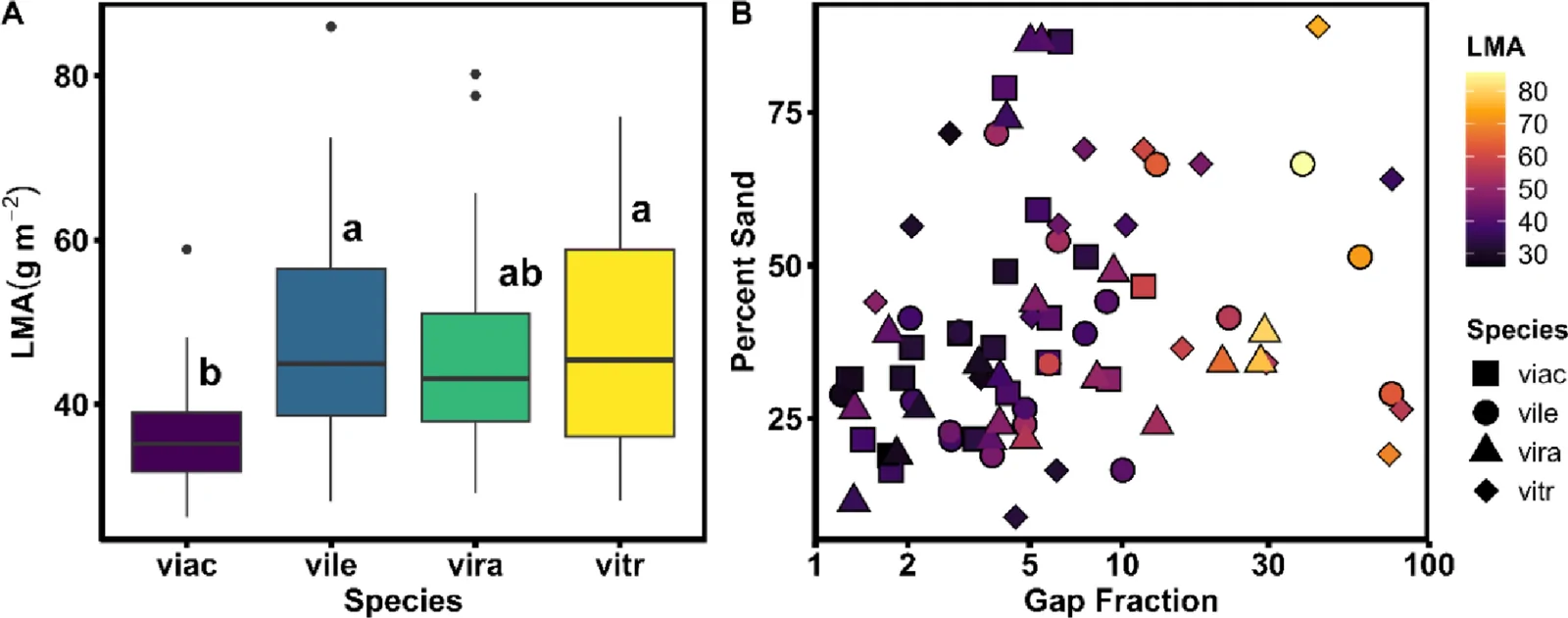
(**A**). Boxplot shows species differences in LMA, with letters above boxplots indicating significant differences. (**B**) Variation in LMA (color gradient) in relation to gap fraction and percent sand. Species abbreviations include the first two letters of both the genus and specific epithet, with ‘viac’ representing *V. acerifolium*, ‘vile’ representing *V. lentago*, ‘vira’ representing *V. rafinesquianum*, and ‘vitr’ representing *V. trilobum*

Overall, the highest performing (highest R_lik_^2^), most parsimonious models tended to be those that included species, gap fraction, and their potential interaction term as fixed effects. Models with only percent sand had less explanatory value and failed to improve model fit (Table 1). Both LMA and D_stem_ displayed significant differences among species, with *V. acerifolium* having significantly lower LMA and higher D_stem_ than *Viburnum trilobum *(Fig. 1a, Sup Table 1). However, the addition of gap faction to models resulted in a modest R_lik_^2^ of 0.4 (Table 1). An R_lik_^2^ of 0.66 highlights the significant effect of gap fraction on LMA beyond significant species and site level effects (Table 1; Fig. 1a and b). There was no significant difference in δ^13^C among species, corroborated by the low R_lik_^2^ values for models including a species term for δ^13^C (Supplemental). Model fit for δ^13^C shows that models that individually include gap fraction have the highest R_lik_^2^ values, explaining 41% and 50% of the variation in δ^13^C (Table 1; Fig. 5a and b).

**Fig. 2:**
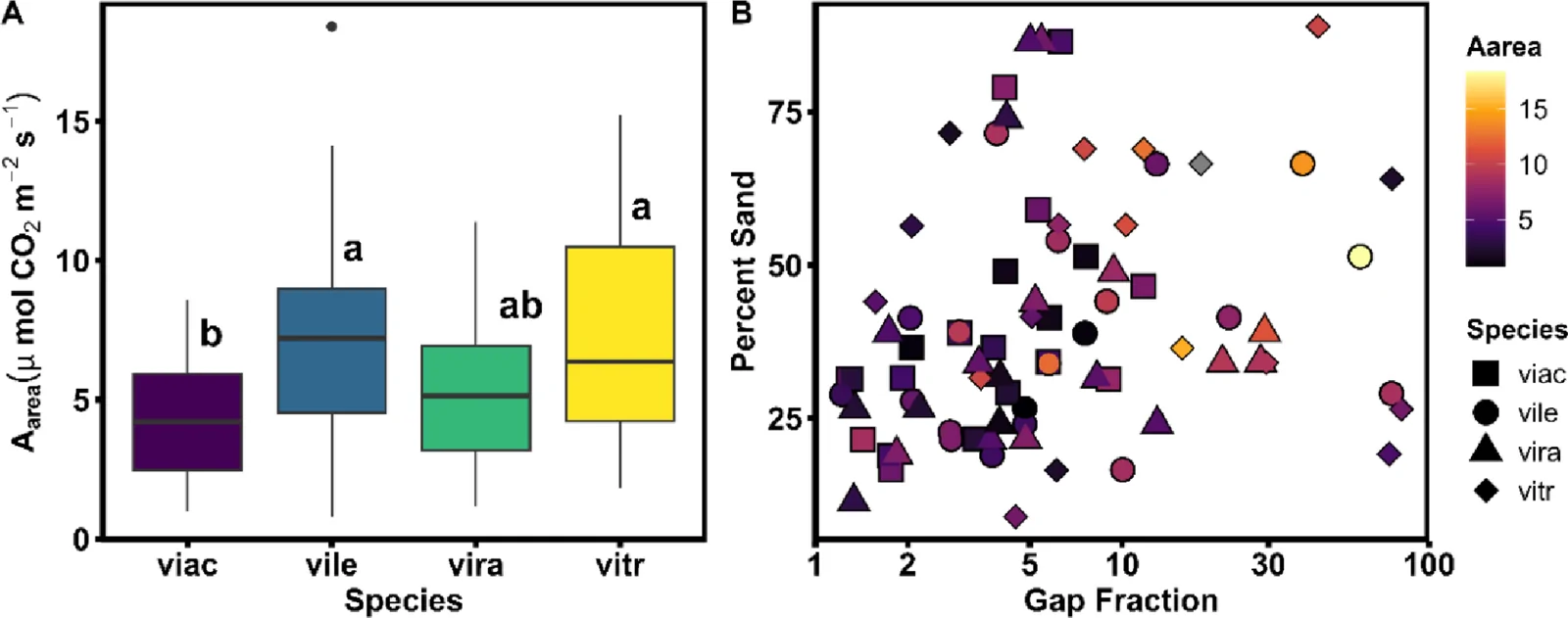
(**A**) Boxplot showing species differences in area-based light-saturated assimilation rate (Aarea). Letters above boxplots indicate significant differences. (**B**) Variation in Aarea (color gradient) in relation to gap fraction and percent sand. Species abbreviations are defined in Fig. 1.

**Fig. 3:**
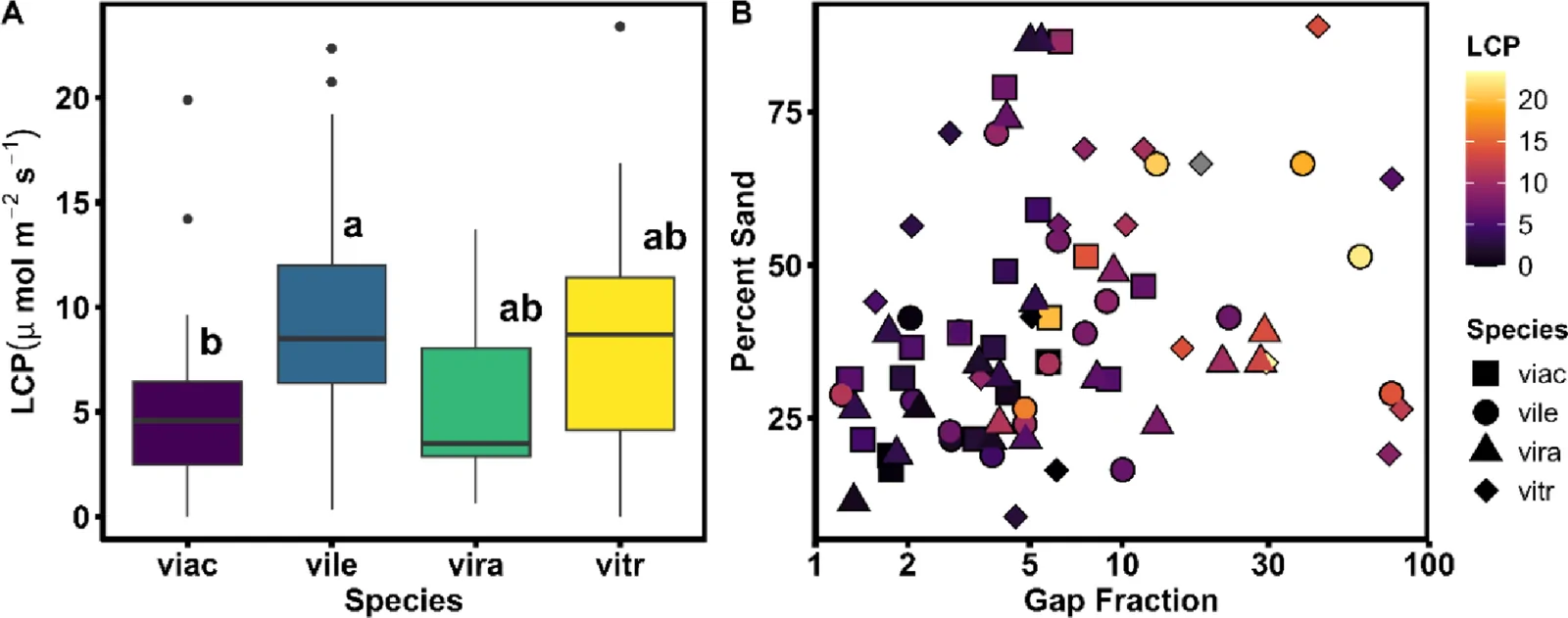
(**A) **Boxplot showing species differences in light compensation point (LCP). Letters above boxplots indicate significant differences. (**B**) Variation in LCP (color gradient) in relation to gap fraction and percent sand. Species abbreviations are defined in Fig. 1.

The best performing models for A_area_ and LCP involved an interaction between species and gap fraction, demonstrating differing species-level positive correlations in gas-exchange on an area basis (Table 1; Figs. 2 and 3 and S4, Sup Table 2). However, all models failed to explain variation in mass-based gas-exchange traits beyond the null model (Table 1). *V. acerifolium* showed significantly lower A_area_ than *V. lentago* and *V. trilobum*, in addition to a significantly lower LCP than *V. lentago* (Figs. 2a and 3a, Supplemental). Models including sand as a fixed effect for gas-exchange traits failed to increase R_lik_^2^ and explain additional variation in traits (Table 1). Only marginal species or environmental trends were identified for K_S_ or K_S, L_, (Table 1, Supplemental) persisting even when a statistical (though validated) outlier of *V. rafinesquianum* with a high K_S, L_ was omitted. However, A_L_A_S_ and Ψ_TLP_ models consisting of a species interaction with gap fraction both demonstrated the highest respective R_lik_^2^ of 0.40 (Table 1). While no significant difference was seen between species in A_L_A_S_, *V. rafinesquianum* had a significantly higher Ψ_TLP_ than *V. trilobum* (Fig. 5a, Sup Table 1).

**Fig. 4:**
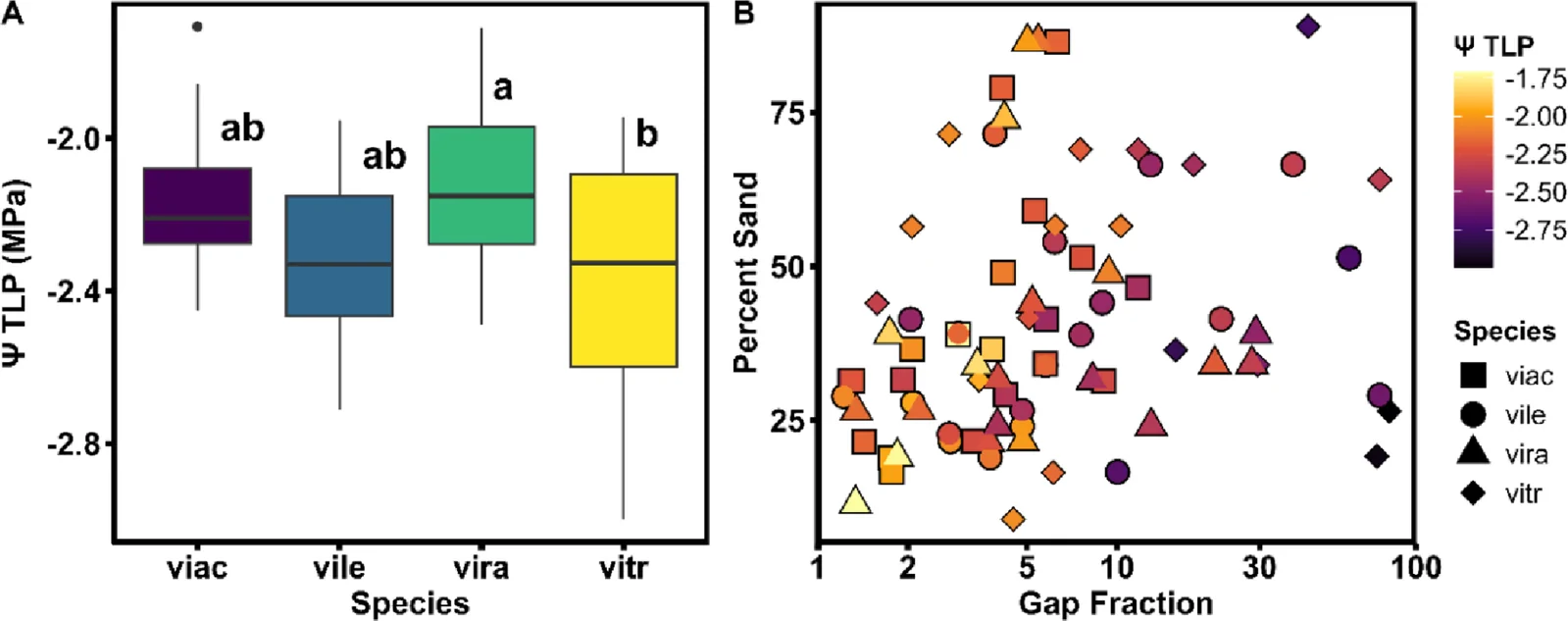
(**A**) Boxplot showing species differences in turgor loss point (Ψ_TLP_). Letters above boxplots indicate significant differences. (**B**) Variation in Ψ_TLP_ (color gradient) in relation to gap fraction and percent sand. Species abbreviations are defined in Fig. 1

Species overlapped substantially in trait space, with *V. trilobum* and *V. lentago* enveloping the smaller trait space of *V. rafinesquianum* and *V. acerifolium*. The first two PC axes explained a combined 52% of the variance within the trait data collected (Fig. 6). PC1 axis was strongly correlated with LMA, K_S_, and Ψ_TLP_, while PC2 was more correlated with constructional traits such as A_L_A_S_, C: N ratios, D_stem_, and δ^13^C (Fig. 6). Broadly, gas-exchange traits were positively correlated, with only *V. acerifolium* showing mostly insignificant correlations, and no traits correlated with R_d_ (Fig. 7). Turgor loss point was negatively correlated with many gas-exchange and construction traits like LMA and δ^13^C (Fig. 7). Overall, conductivity traits and D_stem_ were rarely correlated with other traits, highlighting the low variance and interspecific variation identified in preceding models (Table 1; Fig. 7 Sup Table 1).

**Fig. 5:**
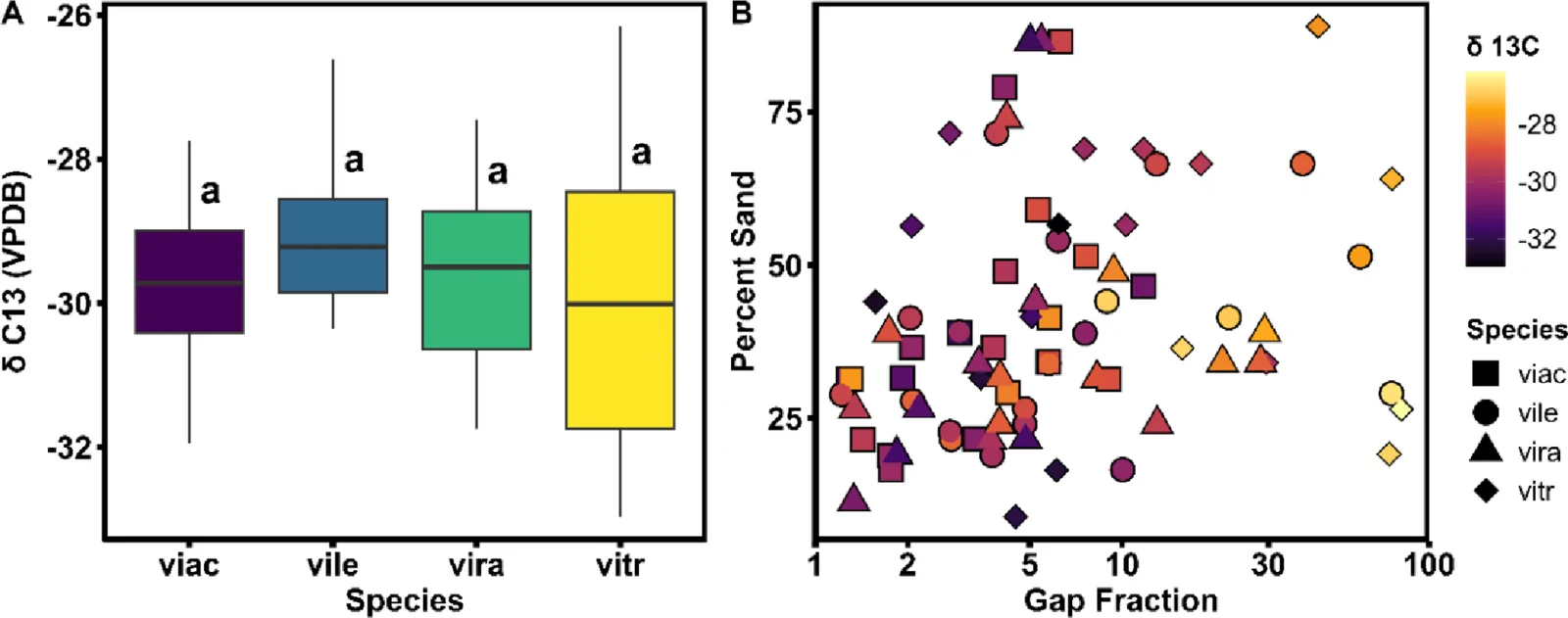
(**A**) Boxplot showing species differences in stable isotope signature (δ^13^C). Letters above boxplots indicate significant differences. (**B**) Variation in δ^13^C (color gradient) in relation to gap fraction and percent sand. Species abbreviations are defined in Fig. 1

**Fig. 6:**
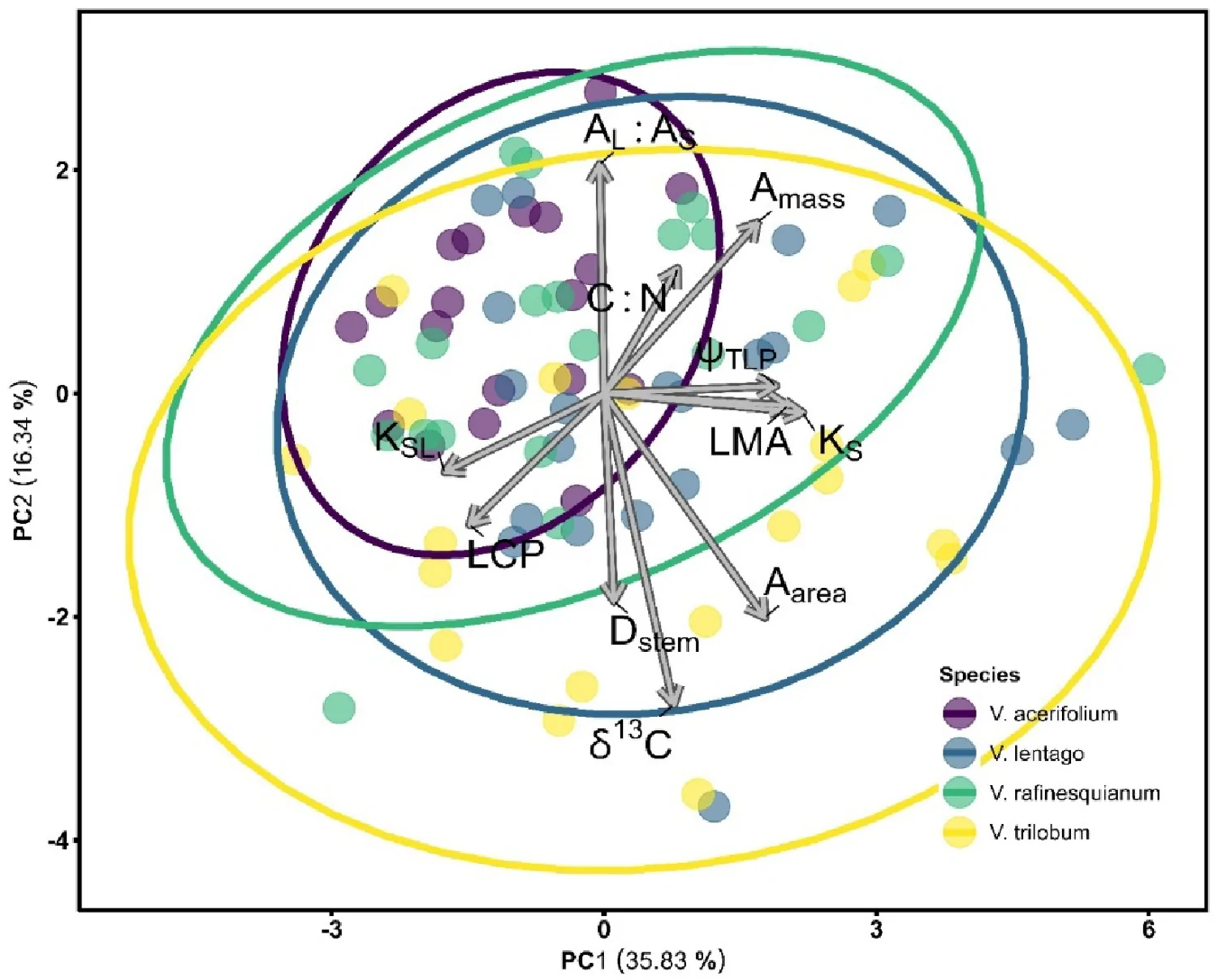
Principal components analysis showing trait space of *Viburnum* congeners, with colors identifying species, ellipses demonstrating 95% confidence level. Parentheses in axes show variance explained by PC1 and PC2, respectively. Traits included in PCA include A_L_:A_S_ (Leaf Area – Sapwood Area ratio), C:N (leaf carbon – leaf nitrogen ratio), A_mass_ (max assimilation rate on a leaf mass basis), Ψ_TLP_ (turgor loss point), KS (maximum stem specific hydraulic conductivity), LMA (leaf mass per area), LCP (instantaneous leaf light compensation point), A_area_ (max assimilation rate on a leaf area basis), D_stem_ (stem density), δ^13^C (heavy carbon isotope signature), and K_S,L_ (leaf area adjusted maximum stem conductivity)

## Discussion

### Significant trait variation explained by environmental gradients

Canopy gap fraction demonstrated stronger correlations with shade tolerance traits than soil sand content. These significant findings expand upon previous studies of physiological impacts of canopy structure in other congeneric systems (Givnish 1988; Givnish et al. 2004), with the notable addition of quantifying negative correlations between gap fraction and Ψ_TLP_. Factors associated with canopy structure in temperate systems significantly influence not only traits related to gas exchange, but also key traits describing leaf-level water status. Significant inter-specific differences in gas-exchange traits, LMA and Ψ_TLP_ also highlight potential adaptive differentiation in these sympatric species (Montgomery and Givnish 2008; Maréchaux et al. 2015) (Supplemental Figures 1-5). In contrast, environmental variables show little impact on hydraulic and stem traits, often shown to be more phylogenetically conserved (Sanchez-Martinez et al. 2020) (Tables 1 and 1b, Supplemental).

**Fig. 7:**
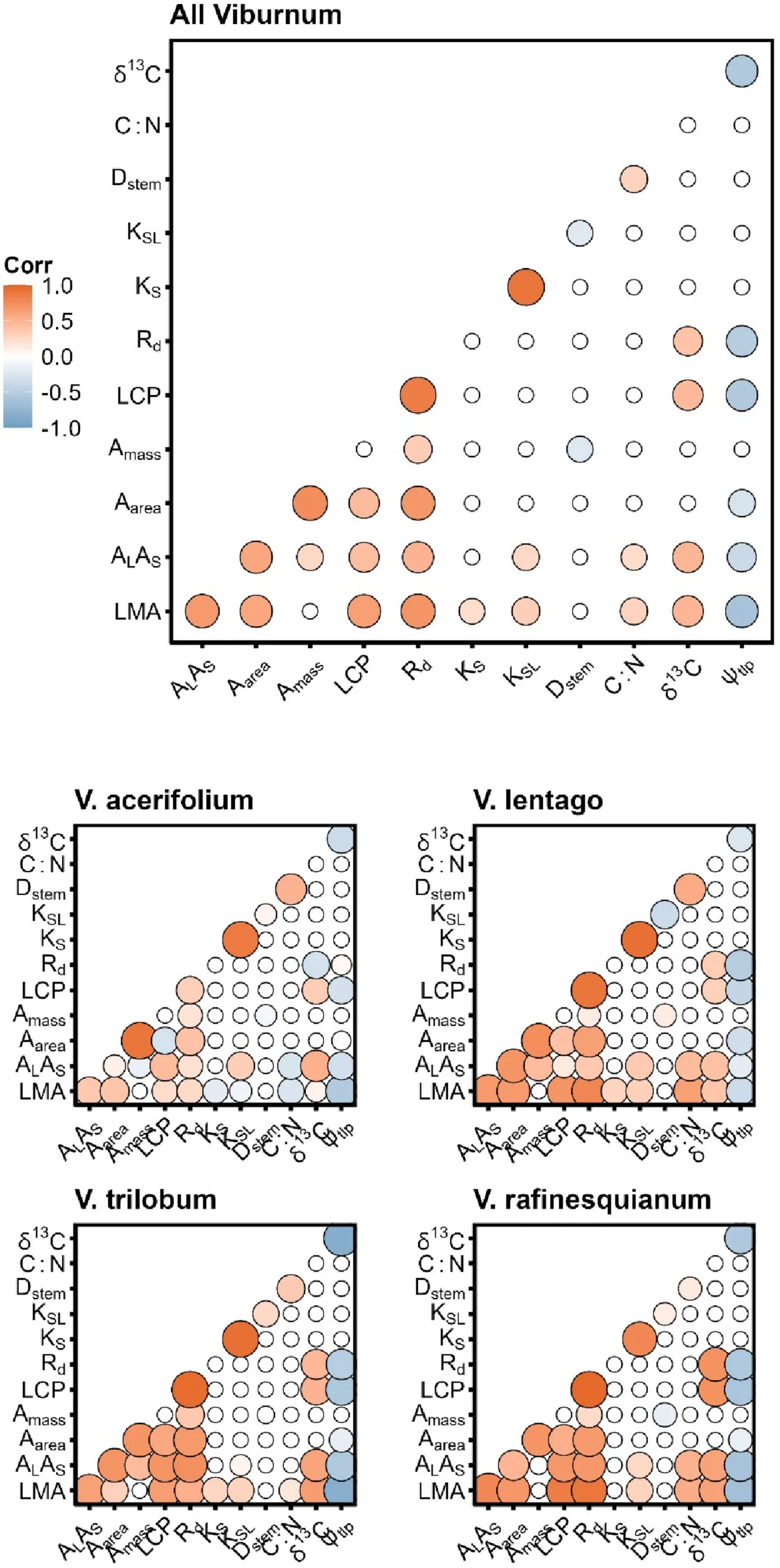
Trait correlations for traits presented in Fig. 6 for all Viburnum and individual species, with only significant positive (red) and negative (blue) relationships denoted by circle size and color intensity. Trait abbreviations are the same as Fig. 6, with the addition of R_d_ (instantaneous dark respiration)

Soil texture explained little trait variation (Table 1). These findings are surprising, given previous work has implicated soil texture as a major driver of heterogeneity in climatically homogenous southern WI forest systems (McCormick and Curtis 1960). Microsite variation in soil texture has also been identified as driving within-stand variation in understory composition in various temperate communities (Leach and Givnish 1999; Meisel et al. 2002). Percent sand ranged from ~ 8% to ~ 88% across our study samples, capturing a large range of soil textures for our trait-environment modelling. Furthermore, trait data were also collected during a prolonged drought period throughout summer 2023, the effects of which are predicted to be greater in sandier soils (Campbell and Norman 1998). Confounding factors such as the depth to water table and the lack of root trait data highlight a limitation of our study in explaining the null result of our trait – soil texture hypotheses.

### Construction traits

Overall, individuals found in shadier environments tended to have lower LMA, consistent with previously identified trends in temperate, broadleaf species (Givnish 1988; Craine and Reich 2005). Stem density varied across species and demonstrated little variation along sand or canopy structure gradients, representing more conserved allocational and construction costs, with *V. trilobum* stem tissue requiring less overall investment (Sup Table 1). Many understory woody species are noted for denser wood and low growth rates, potentially contributing to the interspecific differences seen in *V. acerifolium* when compared to *V. lentago* or *V. trilobum* (Craine and Reich 2005) (Sup Table 1). Notably, we found no evidence of interspecific differences in A_L_A_S_ or mean leaf area, both proposed strategies for increasing whole-plant carbon gain in shade adapted species (Givnish 1988) (Sup Table 1). All species also showed a positive correlation between A_L_A_S_ and increasing gap fraction similar to other deciduous/evergreen subtrees (Rosas et al. 2019; Sack et al. 2006) (Table 1, S3, Sup Table 2). Interestingly, our findings of a positive correlation between light environment and A_L_A_S_ contradict negative correlations found for other deciduous trees (Sack et al. 2006). Our trait syndromes support the contention that more shade adapted species in temperate forests tend to have thinner leaves and dense stem tissue, supporting the characterization of *V. acerifolium* as shade-tolerant when compared to its congeners.

### Gas-exchange

The significant correlations of gas-exchange traits with light environment support our hypotheses and demonstrate significant acclimation across all species to maximize carbon gain (Givnish 1988; Givnish et al. 2004; Craine and Reich 2005) (Figs. 2 and 3, S3). In an understory environment that does not provide consistent irradiance, the reduced metabolic cost of lower R_d_ and LCP buffer the reduced capacity of A_area_. When coupled with lower levels of leaf investment in terms of both LMA, C:N ratios, and A_L_A_S_ (Fig. 1, Sup Table 1), the foliage of understory individuals stands to recoup seasonal investments of carbon into photosynthetic tissue at analogous rates to more robust, open-grown foliage. The lack of distinct differences in A_mass_ among species or along environmental gradients supports this picture of uniform photosynthetic “return on investment.” However, our measurements of instantaneous A_mass_, LCP, and R_d_ may have missed broader seasonal trends in assimilation, light requirements, and respiration that separate the species more fully (Givnish and Montgomery 2014). Our results advance the hypothesis that temperate *Viburnum* display divergent whole-plant compensation points and adaptive crossover along understory gradients of light availability. Controlled greenhouse and/or common garden experiments could more directly test this hypothesis using *Viburnum* as an excellent temperate woody study system.

### Hydraulic adjustment to intermittent light availability

Given the lack of variation in K_S, L_ or K_S_ along environmental gradients, these hydraulic traits do not explain *Viburnum* acclimation or adaptation to varying light or soil texture regimes (Table 1). Additionally, K_S, L_ or K_S_ show no correlation with other stem traits like D_stem_ (Fig. 7). Stem density is an emergent property of wood tissue that can be influenced by multiple traits, including allocation to pith and fibers, conduit wall thickness, and vessel diameter among others (Lachenbruch and McCulloh 2014). Relationships between the capacity to conduct water and the water supplied to a given leaf area do not vary across replicates, despite a positive correlation between A_L_A_S_ and gap fraction (Table 1). Such findings demonstrate that congeners diverge in construction costs of stem tissue and increase the amount of leaf tissue for given stem tissue in gap environments yet maintain the hydraulic support for a given area of photosynthetic tissue. This response is simultaneously coordinated with increased LMA in gap-occupying individuals, resulting in a dense, robust foliage without significantly altered K_S, L_ in gap environments, though there is a slightly positive correlation between K_S_ and K_S, L_ when looking at all species (Table 1; Fig. 7).

Leaf-level hydraulic adjustment is supported by significant trends identified in A_L_A_S,_ Ψ_TLP_, and δ^13^C. Previously derived mechanistic models connecting stem hydraulics to canopy transpiration demonstrate that for woody shrubs, species can adjust A_L_A_S,_ Ψ_leaf_, and stomatal conductance (g_s_) to maximize carbon gain (Whitehead et al. 1984; Buckley and Roberts 2006). In cases of increasingly droughted or water-limited conditions, species must either decrease g_s_ and/or Ψ_leaf_ to offset a given A_L_A_S_ (Buckley et al. 2023) or reduce A_L_A_S_ by dropping leaves (Hoffmann et al. 2011; Marchin et al. 2010). Our results demonstrate an increase in A_L_A_S_ in more open environments (Table 1, S3), necessitating a lower Ψ_leaf_ to maintain hydraulic support and faster reduction of g_s_ as water becomes more limiting.

While more robust mesophyll and palisade parenchyma have been notable in high-light acclimation and net carbon gain, recent work has shown that thicker leaf mesophyll is correlated with higher leaf hydraulic capacitance, with mesophyll conductance showing rapid changes in response to bright light induction (Liu et al. 2022). Leaf water transport and it’s response to rapidly changing Ψ_leaf_ highlight the importance of leaf hydraulic acclimation to dynamic light conditions in forest understories (Givnish 1988; Scoffoni et al. 2023). In addition to more established arguments of the elevated photosynthetic capacity of sun acclimated leaves, our in situ LMA, Ψ_TLP_, and δ^13^C results contribute to developing perspectives of ‘light acclimated’ leaves acting as hydraulic capacitors, buffering against rapid fluctuations in light conditions (Scoffoni et al. 2008; Buckley et al. 2023), though care should be taken when interpreting δ^13^C, since leaf nitrogen significantly varied between species (Sup Table 1). Further experimental work to characterize the extent to which mesophyll photosynthetic sensitivity may be applied to our understanding of the hydraulic demand during rapid variation in irradiance would expand our understanding of acclimation and adaptation to light or shade (Salvi et al. 2021; Smith et al. 2023; Wu et al. 2023).

Notably, Ψ_TLP_ has commonly been gathered on sun-exposed leaves for both standard Scholander pressure-apparatus quantification and contemporary studies using vapor-pressure osmometry (Bartlett et al. 2012; Tyree and Hammel 1972). Given the significant negative effect of gap fraction in Ψ_TLP_ across congeners, sampling only from fully exposed leaves may be systematically overestimating species’ drought tolerance with lower species-mean Ψ_TLP_, especially for temperate or forest species known to occur along gradients of canopy coverage. While Ψ_TLP_ shows promise in characterizing varying drought tolerance among species, variation in Ψ_TLP_ during experimental droughts highlight moderate amounts of acclimation (Bartlett et al. 2014; Maréchaux et al. 2015). While other studies have highlighted the variation of Ψ_TLP_ in relation to drought treatments, habitat, and season, our results identify significant impact of light environment on this increasingly utilized leaf trait (Bartlett et al. 2014; Maréchaux et al. 2020; Eisley and Wolfe 2024). As such, care should be taken when attempting to characterize Ψ_TLP_ in environments with heterogenous light availability, especially for species adapted to low-light habitats.

While we demonstrate significant variation among species for some traits, the broad overlap in the trait syndromes of these four *Viburnum* species and their phylogenetic distance within the genus suggests a pattern of convergent evolution. Recent work supports the origin of *Viburnum* in warm temperate forests in southeast Asia, with each species we studied descending from a distinct entries into North America (Landis et al. 2021). Each lineage also demonstrates adaptation to the cold-temperate climates throughout the Mid-Late Miocene, including notable shifts towards deciduous and lobed leaves in line with support-supply hypotheses of gross leaf morphology (Givnish 2002; Schmerler et al. 2012; Givnish and Kriebel 2017; Landis et al. 2021). Our findings provide additional physiological evidence of trait convergence for these distantly related congeners in cold-temperate deciduous understories. while also serving as a modal system for further studying the integration of leaf physiology into the support-supply hypothesis (Givnish and Kriebel 2017).

Overall, the four *Viburnum* species varied significantly in terms of gas-exchange and anatomical traits both among species and along gradients of increasing gap fraction in the canopy, while stem hydraulic traits demonstrated no relationship with canopy structure. Soil texture had little significant impact on any of the assessed traits. Strong trait trends in photosynthetic capacity, δ^13^C, and especially Ψ_TLP_ highlight an acquisitive trait syndrome in gap-occupying individuals regardless of species, with *V. acerifolium* appearing more functionally constrained to denser canopies than other congeners. Overlapping trait values among species highlight limited differentiation in trait syndromes, but a broader study considering relative growth rates, allometry, reproductive success, and whole-plant compensation point might shed light on broader aspects of niche conservatism and adaptation. Future experimental studies using varying levels of irradiance could also better clarify trait coordination between hydraulic and photosynthetic traits observed in gap-occupying individuals. For all species, strong coordination in Ψ_TLP_ and δ^13^C with increasing gap fraction and no significant variation in K_S, L_ alludes to potential physiological importance of buffering against low Ψ_leaf_ with limited hydraulic support in high or fluctuating light environments. Our findings open new opportunities for studying the impacts of sunflecks and gap dynamics on leaf hydraulic traits and light acclimation, especially regarding hydraulic dynamics and mesophyll photosynthetic sensitivity in forest gap environments. More broadly, our results also emphasize careful consideration of sampling protocols in characterizing Ψ_TLP_ at the species level using only sunlit leaves.

## Supplementary Information

Below is the link to the electronic supplementary material.

Supplementary Material 1

## Data Availability

All trait and environmental data collected for this publication can be found here: http://datadryad.org/share/XcLAcwNHiWfqC98jwRecJzv4HANrQmGlSbKxutbGM-s.
