# Supplementary material for: Co-occurring congeners coordinate drought and shade tolerance traits along gradients of canopy openness
Source: Oecologia. 2026 Aug 10;208(9):113. doi: 10.1007/s00442-026-05950-1 (PMC13457493; doi:10.1007/s00442-026-05950-1)
Supplement: Supplementary file 1 [file 442_2026_5950_MOESM1_ESM.docx]

**TITLE: Co-occurring congeners coordinate drought and shade tolerance traits along gradients of canopy openness**

**AUTHORS:** Sam W. Anderson^1*^, Jackson Cramer^1^, Katherine A. McCulloh^1^

**Author Affiliations**

1. University of Wisconsin – Madison Botany Department; 430 Lincoln Dr, Madison, WI 53706

* Corresponding Author – [swanderson5@wisc.edu](mailto:swanderson5@wisc.edu); 715-529-1915; <https://orcid.org/0009-0005-6016-9873>

**Supplemental Information**

S1: From Landis *et al 2021* (Figure 2)¸showing the four congeners of interest in relation to 164 other taxa within *Viburnum.* Syst Biol, Volume 70, Issue 1, January 2021, Pages 67–85, <https://doi.org/10.1093/sysbio/syaa027>

S2: A map of the ten sites where *Viburnum* samples were collected summer 2023. Inset shows the larger geographical context of the upper Great Lakes region of central North America.

S3: Predicted models of significant trait variation along gradients of gap fraction shown in Table 1a and 1b, including a species level interaction. Models follow R package *lmer4* syntax lmer(trait ~ loggap * species + (1|site)) for A) LMA, B)A_area_, C) A_L_A_S_ (m^2^:cm^2^), D) instantaneous LCP, E) Ψ_TLP_, and F) δC_13._

Sup Table 1: Trait means for each species, accompanied by a compact letter display of significance for ANOVA and post-hoc Tukey’s Honest Significant Difference.

| Trait | Unit | *V. acerifolium* | *V. lentago* | *V. trilobum* | *V. rafinesquianum* |
| --- | --- | --- | --- | --- | --- |
| A_L_A_S_ | m^2^:cm^2^ (log transformed) | 0.099a | 0.155a | 0.145a | 0.176a |
| A_area_ | µmol m^-2^ s^-1^ | 4.253b | 7.496a | 7.103a | 5.468ab |
| A_mass_ | µmol g s^-1^ | 0.107a | 0.139a | 0.162a | 0.11a |
| C | % | 46.324a | 46.131a | 46.484a | 44.785a |
| C:N | unitless | 26.086a | 24.13a | 26.447a | 26.88a |
| δ^13^C | VPDB (%) | -29.746a | -28.963a | -29.89a | -29.672a |
| Height | m | 1.61b | 2.375a | 1.953ab | 1.735b |
| K | g mm s^-1^ MPa^-1^ | 30.992b | 48.246ab | 64.47a | 39.353ab |
| K_S_ | g mm^-1^ s^-1^ MPa_-1_ | 0.987a | 1.158a | 1.51a | 1.118a |
| K_S,L_ | g m^-1^ s^-1^ MPa^-1^ | 1.399E-3a | 9.98E-4a | 1.129E-3a | 1.548E-3a |
| LCP | PAR | 5.318b | 9.873a | 8.595ab | 5.509ab |
| Leaf Area | cm^2^ | 34.925a | 34.78a | 31.587ab | 23.014b |
| LMA | g m^-2^ | 36.399b | 49.075a | 48.067a | 46.768ab |
| N | % | 1.804ab | 1.947a | 1.799ab | 1.696b |
| N^15^ | Air (%) | -4.57c | -2.382ab | -1.493a | -2.915b |
| D_sapwood_ | g cm^-3^ | 0.665a | 0.559bc | 0.506c | 0.61ab |
| D_stem_ | g cm^-3^ | 0.578a | 0.517b | 0.433c | 0.553ab |
| Ψ_TLP_ | MPa | -2.173ab | -2.309ab | -2.375b | -2.119a |

Sup Table 2: P-values for Type II Wald Chi-Square Test (function *Anova* package *car*) for models displayed in S3. Bold p-values denote significant fixed effects and interactions, while italicized values are marginally significant.

**Analysis of Deviance Table (Type II Wald chi-square tests)**
